# Defect Detection Method of Carbon Fiber Unidirectional Band Prepreg Based on Enhanced YOLOv8s

**DOI:** 10.3390/s25092665

**Published:** 2025-04-23

**Authors:** Weipeng Su, Mei Sang, Yutong Liu, Xueming Wang

**Affiliations:** 1 School of Precision Instrument and Opto-Electronics Engineering, Tianjin University, Tianjin 300072, China; weipengsu@tju.edu.cn (W.S.); liuyt0226@tju.edu.cn (Y.L.); 2 Key Laboratory of Opto-Electronics Information Technology, Ministry of Education, Tianjin 300072, China; 3 Composite Test Technology Center, AVIC Composite Corporation Ltd., Beijing 101300, China

**Keywords:** carbon Fiber unidirectional band prepreg, defect detection, deeplearning, YOLOv8s, GAM, DLKA

## Abstract

To address the challenges in existing carbon fiber prepreg surface defect detection processes, specifically difficulties in small target detection and inaccuracies in elongated defects with large aspect ratios, this study proposes an enhanced YOLOv8s-based models for defect detection on the surface of carbon fiber unidirectional band prepreg. The proposed model respectively integrates two attention mechanisms, a Global Attention Mechanism (GAM) and a Deformable Large Kernel Attention (DLKA) Mechanism, into the architecture of YOLOv8s, which is a lightweight version of YOLO optimized for speed. The attention mechanisms are inserted between the backbone network and the detection head, respectively, to enhance feature extraction before target localization. The YOLOv8s-GAM model achieves a mean average precision (mAP@0.5) of 84.4%, precision of 79.3%, and recall of 78.3%, while the YOLOv8s-DLKA model shows improved performance with mAP@0.5 of 86.4%, precision of 82.4%, and recall of 80.2%. Compared with the original YOLOv8s model, these two modified models demonstrate improvements in mAP@0.5 of 1.6% and 3.6%, precision gains of 0.9% and 4.1%, and recall enhancements of 1.4% and 3.6%, respectively. These models provide technical solutions for precise defect identification on the surface of carbon fiber unidirectional band prepreg.

## 1. Introduction

In the rapidly developing modern industry, the performance of materials directly determines product quality and the expansion of application domains. Carbon fiber unidirectional band prepreg, as a high-performance material, has gained prominence in aerospace, automotive manufacturing, sporting equipment, and other high-end industries due to its high strength, low density, and exceptional corrosion resistance. In the aerospace field, carbon fiber reinforced composites can reduce the weight of aircraft while enhancing fuel efficiency and flight performance. In automotive manufacturing, they contribute to vehicle lightweighting, thereby reducing energy consumption and improving handling [1,2,3].

Carbon fiber prepreg is an intermediate material for composite materials. During its production process, various surface defects inevitably arise due to manufacturing processes and carbon fiber raw material quality [4,5,6], and conventional manual inspection methods exhibit significant limitations for these detections. Firstly, manual inspection is highly inefficient. As production scales increase, human visual inspection can hardly meet mass production demands, severely slowing production workflows. Secondly, detection accuracy cannot be guaranteed. Prolonged watching tasks result in visual fatigue, lead to missed or erroneous detection of subtle defects like fiber clusters and splits—particularly in complex industrial environments where lighting variations and background interference further impede judgment consistency. Thirdly, manual inspection incurs substantial costs requiring large teams of skilled inspectors, which need not only high labor expenses but also time-consuming training investments. Moreover, personal subjectivity between inspectors due to varying experience levels and evaluation criteria results in inconsistent and unreliable detection outcomes. When human visual inspections fail to detect defects in carbon fiber pre-impregnated materials (prepregs), the undetected surface defects may propagate into the subsequent composite material molding process. This can lead to defective components and, in severe cases, result in scrapping of the finished parts, causing significant cost wastage. Therefore, the development of artificial intelligence (AI)-based automated detection technology for surface defects in carbon fiber prepregs is imperative to address these challenges and ensure product quality and production efficiency.

AI-based object detection algorithms have found extensive applications in diverse domains. Yu et al. proposed a novel hybrid framework for automated delamination detection of bridge decks based on ground penetrating radar (GPR), a mature technique used to locate subsurface deterioration or damage in bridges. This framework incorporates synchrosqueezed wavelet transform (SSWT), a convolutional neural network (CNN), transfer learning, and metaheuristic optimization methods, achieving a prediction accuracy exceeding 94% [7]. Tang et al. proposed ConTriNet, a robust confluent triple-flow network framework adopting a “divide-and-conquer” strategy. The framework utilizes a shared encoder and dedicated decoders to separately handle subtasks of exploring modality-specific and modality-complementary information, thereby enhancing the final saliency map prediction performance. ConTriNet consistently outperforms state-of-the-art methods in both common and challenging scenarios, demonstrating excellent performance even when dealing with incomplete modality data [8]. Jiang et al. proposed an efficient lightweight damage recognition model named YOLOMF, which is constructed by introducing MobileNetv3 and fused inverted residual blocks into You Only Look Once v4 (YOLOv4). YOLOMF demonstrates excellent multi-damage recognition capabilities for concrete bridges under various field-of-view sizes and complex environmental conditions, achieving a detection speed of 85 frames per second [9].

With growing demands for automated carbon fiber prepreg surface defect detection, several methods have been applied in this field. Liu et al. proposed a novel model called CFP-SSD using an enhanced Single Shot MultiBox Detector (SSD) for surface defect recognition on carbon fiber prepregs. They established a machine vision-based defect detection platform. The improved ResNet50 backbone in the CFP-SSD model enhanced feature extraction efficiency. By designing a multi-scale fusion module for remote contextual feature extraction to effectively integrate shallow and deep information, the CFP-SSD achieved an 86.63% average precision and a 47 fps detection speed [10]. Wen et al. developed an improved YOLOv7 algorithm to boost performance in carbon fiber prepreg surface defect detection. The introduction of BiFormer attention mechanisms enabled better focus on small-target defects, improving feature discriminability. The Path Aggregation Feature Pyramid Network (PAFPN) in the neck layer was replaced by an Asymptotic Feature Pyramid Network (AFPN) to strengthen feature fusion, retain more semantic information, and refine multi-scale feature integration. The adoption of WIoU (Wise Intersection over Union) instead of CIoU (Complete Intersection over Union) as the bounding box regression loss function enhanced sensitivity to small targets and accuracy in bounding box prediction. Compared to the baseline YOLOv7 model, the modified YOLOv7 achieved a 10.5% increase in mAP@0.5 and a frame rate improvement of 14 fps [11]. Sun et al. introduced a lightweight, edge-computing-friendly defect detection model based on improved YOLOv5 for industrial components. Integrating coordinate attention mechanisms for improved feature extraction, deploying a Bi-directional Feature Pyramid Network (BiFPN) to reduce false negatives/positives for small targets, and adding a Transformer detector to enhance recognition of challenging samples, their model increased recall by 5.3% to 91.6%. Inference speed approached 95 fps with a 2.4% improvement in mAP@0.5 [12]. Zhang et al. proposed an enhanced YOLOv5-based model named Metal-YOLO, incorporating a Cross-layer Feature Enhancement Connection (CFEC) to strengthen representation of complex small defects and reduce false negatives. The integration of adaptive attention modules minimized background interference, while the Efficient Intersection over Union (EIoU) loss function significantly improved bounding box localization accuracy. Their model achieved a 74.1% recall and a 78.3% mAP@0.5, surpassing the baseline YOLOv5s by 5.0% and 4.1%, respectively [13].

Although YOLO [14,15,16,17,18,19,20,21,22] (You Only Look Once) is an efficient object detection framework with high speed and accuracy, existing implementations show deficiencies in detecting small targets and defects with large aspect ratios in carbon fiber unidirectional band prepreg surface inspection scenarios. Practical deployments also experienced false positives, missed detection, and redundant detection.

To address these issues, our study incorporates both a Global Attention Module (GAM) [23] and a Deformable Large Kernel Attention (DLKA) Mechanism [24] between the backbone network and detection head of YOLOv8s. The GAM applies global feature weighting to enhance critical feature extraction capabilities, while DLKA dynamically allocates attention to localized regions for precise small-defect characterization. These improvements aim to comprehensively advance the performance of YOLOv8s in carbon fiber unidirectional band prepreg surface defect detection, enabling rapid and accurate identification of defects. This provides manufacturers with a robust quality inspection tool to support the high-quality development of carbon fiber unidirectional band prepreg industries.

## 2. Dataset and Methodology

### 2.1. Dataset

**Figure 1:**
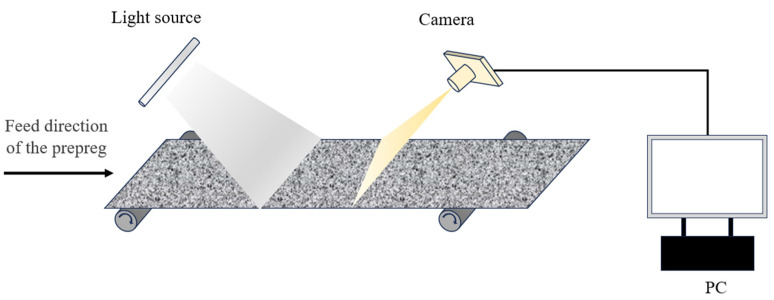
Image acquisition system.

**Figure 2:**
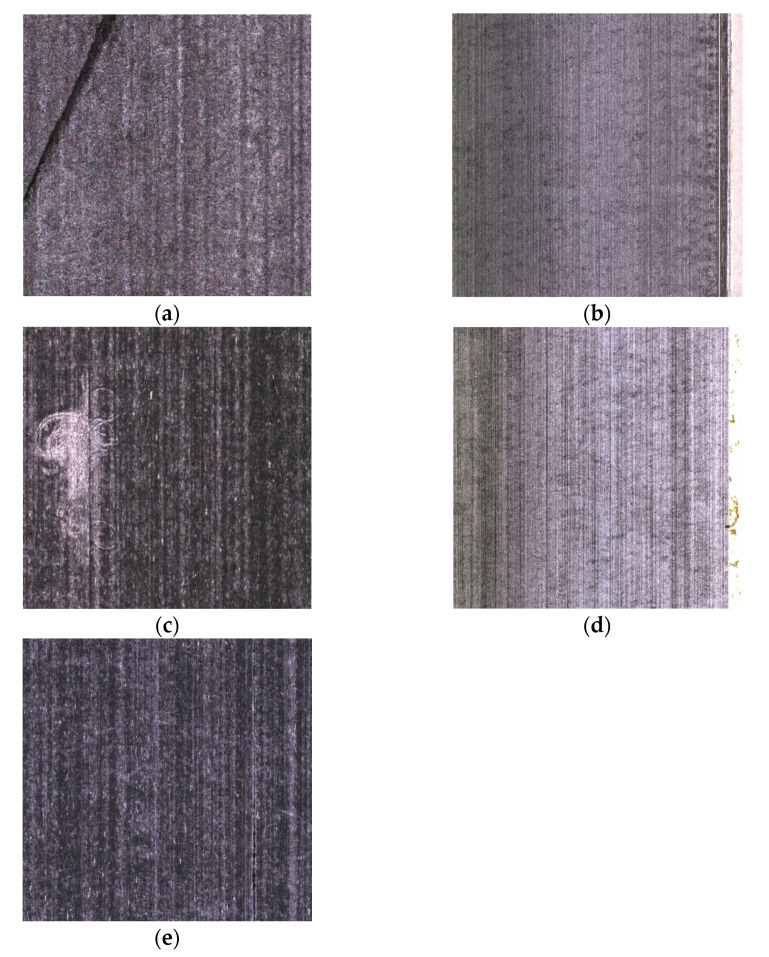
Surface defect categories in prepreg: (**a**) Wrinkles, (**b**) fiber splits, (**c**) fiber tangles, (**d**) resin-rich, (**e**) resin-poor.

The dataset used in this study was collected from actual industrial carbon fiber prepreg production sites in AVIC composite corporation. Figure 1 illustrates the structure of the image acquisition system. The image acquisition system consists of cameras, white light sources, and a carbon fiber prepreg conveyor production line. The prepreg continuously moves along a specific direction on the conveyor belt, while the cameras capture and transmit images to the computer for processing. To train the model, a large number of sample data were required. As shown in Figure 2, the dataset contains 4300 images with a resolution of 4096 × 4000 pixels, divided into training, validation, and test sets in an 8:1:1 ratio. Based on the characteristics of carbon fiber prepreg surface defects, the dataset was manually classified into five categories: fiber tangles (mt), fiber splits (ff), wrinkles (zz), resin-rich (fz), and resin-poor (pj). An in-depth analysis of the carbon fiber prepreg surface defect sample images in Figure 2 reveals that they exhibit a vertically oriented periodic texture pattern across the entire image background, which provides a critical basis for subsequent defect analysis.

Fiber tangles are small agglomerations formed during the rolling process of carbon fiber prepregs due to excessive surface hairs and mechanical friction causing fiber entanglement. These tangles show significant morphological and structural differences from background textures, with their clustered shapes enabling relatively easy identification through contrasting distribution patterns. Fiber splits occur when yarn-splitting devices fail to distribute yarns evenly, manifesting as thin variably sized linear gaps aligned with background textures. Their alignment similarity poses detection challenges through disguising detection through directional resemblance. Wrinkle defects emerge from uneven pressure or tension distribution during yarn rolling and stacking, creating localized folds that substantially compromise material mechanical properties. Resin-rich (yellow areas) result from excessive resin impregnation during manufacturing, while resin-poor (black areas) are caused by insufficient resin coverage. The former, often concentrated at sample edges due to resin flow patterns, alters chemical composition and interfacial bonding. The latter reduces structural integrity and strength through incomplete fiber–resin adhesion, inducing potential displacement under load with critical safety implications.

The dataset was annotated using the LabelImg tool (TzuTa Lin, Taipei City, China), generating label files in .txt format.

### 2.2. Enhanced YOLOv8s Model with Attention Mechanism

#### 2.2.1. YOLOv8 Model

**Figure 3:**
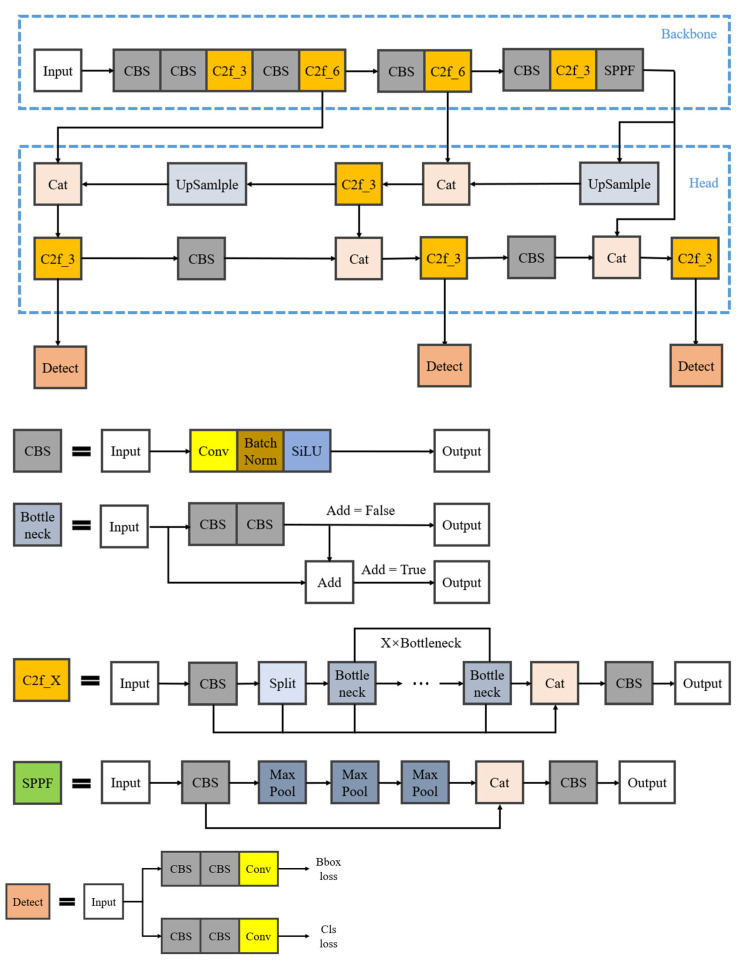
YOLOv8 network architecture diagram (Legend: Split = Slicing operation; Conv = Convolution operation; BatchNorm = Batch normalization operation; SiLU = Activation function; MaxPool = Maximum pooling operation).

In the field of object detection, the YOLO algorithm has emerged as a critical methodology due to its real-time performance, single-stage detection approach, multi-scale feature fusion, unique anchor box design, and multi-task learning framework, achieving efficient and accurate target detection. The following sections detail the improvements to YOLOv8 across three core components: backbone and neck networks, detection head, and loss function. Its network architecture is illustrated in Figure 3.

In the backbone network aspect, YOLOv8 adopts the C2f (cross-stage partial bottleneck with two convolutions) module. The C2f module can organically combine high-level features with contextual information, thereby improving detection accuracy. At the same time, this module adjusts channel numbers for models of different scales, thus significantly enhancing model performance. The neck network plays a crucial role in the overall architecture by effectively bridging the gap between feature representations output by the backbone network and predictions from the head network.

The detection head in YOLOv8 adopts a state-of-the-art decoupled architecture that separates feature learning for object localization and classification. This structural decoupling enhances the capability of the model to process multi-task detection tasks efficiently by optimizing distinct subnetworks for different prediction branches, thereby improving computational efficiency and cross-task adaptability.

The loss function calculation of YOLOv8 is divided into two branches, classification loss and regression loss. For classification loss computation, the binary cross-entropy (BCE) function is adopted; while the regression loss combines distribution focal loss (DFL) and CIoU. This combination makes prediction boxes fit ground truth boxes better, further improving the accuracy of target detection.

The adaptability of the YOLOv8 model to carbon fiber prepreg surface defect datasets manifests in the deep alignment between its network architecture and defect characteristics. The backbone network learns vertical periodic texture patterns through lightweight convolutional layers while effectively suppressing background interference using large-receptive-field pooling; the PAFPN [25] (Progressive Asymmetric Feature Pyramid Network) feature pyramid achieves multi-scale feature fusion, enabling both capture of fine edge features in fiber bundles and perception of global deformation in wrinkles; the dynamic anchor mechanism optimizes detection strategies based on edge distribution characteristics of resin-rich and resin-poor defects; the decoupled head design simultaneously outputs defect locations and category information, meeting industrial quality inspection requirements. The texture-aware attention layer suppresses background noise, data augmentation strategies improve training effectiveness for edge defect samples, and the multimodal fusion architecture enhances recognition capability for defect regions. These designs enable YOLOv8 to accurately locate various defects in complex backgrounds, fully meeting the practical requirements of carbon fiber prepreg quality control.

#### 2.2.2. GAM

**Figure 4:**
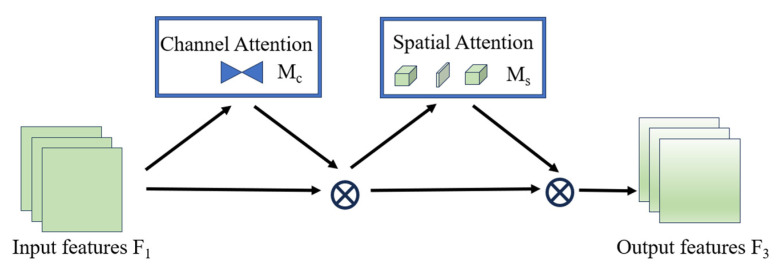
GAM structure.

Traditional attention mechanisms suffer from information reduction and dimension separation, leading to their utilization of limited receptive field visual representations. In this process, they lose global spatial-channel interactions. The GAM is a global attention mechanism spanning the spatial-channel dimension that improves deep neural network performance by reducing information dispersion and amplifying global interaction representations. Its structure is shown in Figure 4, where the two parts represent the channel attention module and spatial attention module, respectively.

**Figure 5:**
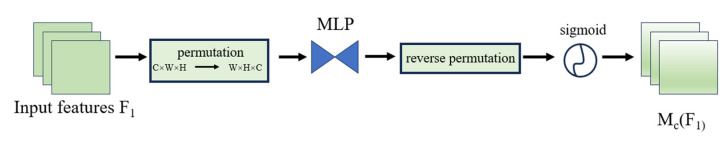
Channel Attention Submodule structure.

The Channel Attention Submodule structure (shown in Figure 5) employs a 3D permutation to preserve information across three dimensions. It then amplifies cross-dimensional channel-space dependencies using a two-layer MLP (Multi-Layer Perceptron).

**Figure 6:**
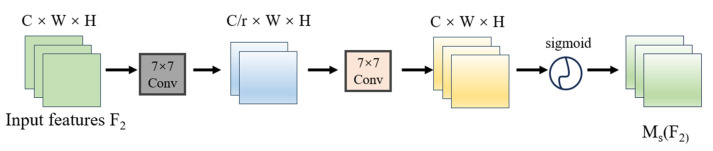
Spatial Attention Submodule structure.

In the Spatial Attention Submodule structure (illustrated in Figure 6), spatial information fusion is achieved through two convolutional layers to emphasize spatial correlations. Notably, the GAM adopts the same reduction ratio r as the Bottleneck Attention Module (BAM) [26] but eliminates the pooling operations. While max-pooling of the BAM leads to information reduction and consequent negative impacts, the GAM removes pooling to better preserve feature map integrity.

This design optimizes computing efficiency while maintaining attention effectiveness. However, the spatial-channel attention framework may still impose significant parameter overhead in certain implementations.

YOLOv8, although it demonstrates superior overall performance compared to previous versions, shows limitations in handling targets like fiber splits due to insufficient global contextual information, making it difficult to accurately capture critical target features. To address this issue, the GAM is introduced.

By weighting global features, the GAM enhances the expressive capacity of target regions during the phase after feature extraction and before object detection, enabling the model to ignore background interference. By intensively focusing on critical target regions, the GAM improves detection accuracy for elongated objects in complex scenarios, particularly for fiber split defects with large aspect ratios. So, it can reduce false positives and missed detections while maintaining precision.

In carbon fiber prepreg surface defect detection, the GAM everages its cross-dimensional global interaction capabilities and forms deep alignment with defect characteristics through unique adaptive processes. For irregular agglomerations of fiber tangles and longitudinal patterns of fiber splits, the spatial attention module focuses on localized detailed features to precisely capture fibrous tangling patterns at the edges of tangles and the linear continuity of splits. The channel attention module amplifies color contrast features of resin-rich and resin-poor defects, effectively distinguishing abnormal yellow or black regions from background textures. For global deformation patterns of wrinkling defects, the GAM global interaction mechanism integrates structural changes across regions and overcomes the limitation of local features to achieve a holistic characterization of wrinkling morphologies.

In complex backgrounds, the GAM suppresses redundant responses from periodic textures, significantly enhancing detection sensitivity for fiber splits and similar targets. Concurrently, it strengthens correlations among multiscale defect features, providing enhanced defect recognition capabilities for industrial quality inspection.

By inserting the GAM between the backbone network (which extracts initial features from input images) and the detection head (responsible for final detection results), the mechanism injects global contextual information post-feature extraction and pre-detection. This allows the model to better capture critical features across the entire image, improving its robustness in handling complex scenes and long-range contextual dependencies.

#### 2.2.3. Deformable Large Kernel Attention

**Figure 7:**
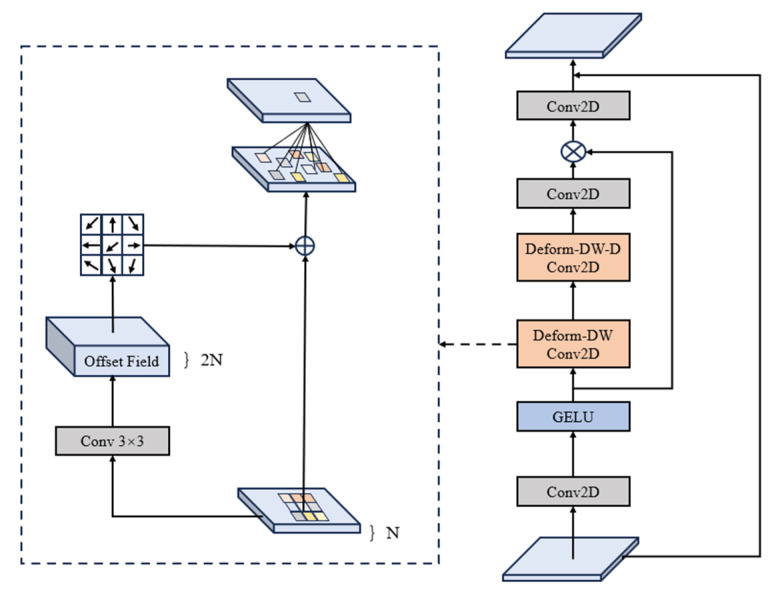
Deformable Large Kernel Attention module.

Deformable Large Kernel Attention (DLKA) is a specially designed attention mechanism for visual tasks, aiming to enhance the flexibility and performance of the model in handling objects of varying sizes and shapes. Drawing inspiration from conventional attention mechanisms, it introduces a deformable kernel to strengthen adaptability to local features. Figure 7 illustrates the schematic diagram of the DLKA module.

Unlike static convolutional filters, DLKA employs deformable kernels that dynamically adjust their spatial configuration during attention computation. This geometric adaptability enables precise feature extraction across objects with varying shapes and scales, particularly enhancing sensitivity to elongated or irregular defects. By replacing conventional small kernels with expanded receptive fields, DLKA efficiently captures long-range dependencies and global contextual patterns. This design addresses the limitations of localized feature extraction in traditional convolutions, making it robust for complex defect distributions. The mechanism integrates multi-scale feature maps through weighted attention maps derived from deformable kernel outputs. This strategy amplifies defect-relevant regions while suppressing background noise, achieving synergistic integration of local details and global semantics. Dynamic kernel deformation adapts to defect geometries without manual parameter tuning, improving generalization across diverse defect typologies. The expanded field of view induced by large kernels concurrently preserves spatial resolution and models defect-environment interactions, providing a critical mechanism for identifying subtle anomalies in complex visual patterns. Multi-scale fusion preserves discriminative features across resolution hierarchies, enabling consistent performance under varying defect sizes.

The applicability of DLKA in carbon fiber prepreg surface defect detection is reflected in its deep alignment with defect characteristics through dynamic deformable large kernel attention mechanisms: Deformable kernels adaptively adjust convolutional kernel shapes to precisely capture the irregular agglomerations of fiber tangles and the elongated directional patterns of fiber splits; the large kernel design expands the receptive field, effectively capturing global deformation features of wrinkling defects; weighted feature fusion mechanisms highlight regions with color contrast (e.g., resin-rich and resin-poor areas) while suppressing background periodic texture interference; multiscale feature fusion architecture balances the capture of fine details in small defects and the global understanding of larger scale defects; and dynamic attention further suppresses background texture responses to enhance recognition of subtle defects. These capabilities allow DLKA to handle the diverse morphologies and multiscale surface defects in carbon fiber prepregs under complex backgrounds with unparalleled flexibility and robustness compared to traditional models.

In order to reduce the false detections of the model, which means to minimize the possibility of false negatives and improve the accuracy of the model, the GAM and DLKA achieve this goal through the following mechanisms, respectively:

Global Information Capture: The GAM captures global information through techniques such as global pooling, enabling the model to comprehensively understand the content of the image. This helps to avoid missing the detection of targets due to the lack of local information and is conducive to reducing false negatives.

Dual Attention Mechanism: The GAM combines channel and spatial attention and emphasizes the interaction among channels, spatial height, and spatial width through 3D arrangement and MLP. This enables the model to more accurately locate and identify targets, enhances the ability to detect targets of different scales and positions, and reduces the probability of false negatives. For example, when detecting multiple objects of different sizes in a complex background, the dual attention mechanism can assist the model in better focusing on the features of each object.

Information Retention and Enhancement: The GAM with the pooling operation removed can better retain the information in the feature maps, enriching the feature representation. Meanwhile, through a weighting method, it ensures that important information is retained during the feature transmission process, allowing the model to make judgments using more complete information in subsequent processing and reducing false negatives caused by information loss.

Deformable Large Kernel: The deformable large kernel of DLKA can adaptively adjust the size and shape of the receptive field, which can better adapt to targets of different shapes and sizes. For some irregular or non-standard shaped targets, the deformable large kernel can more flexibly cover the target area and extract more comprehensive features, thereby reducing false negatives.

Guidance of the Attention Mechanism: As an attention mechanism, DLKA guides the model to focus on the more important regions in the image and suppresses the interference of irrelevant information. When detecting targets, it can help the model concentrate its attention on the regions where targets are likely to exist, improving the detection accuracy of targets and reducing false negatives. For instance, in an image containing multiple objects, DLKA enables the model to focus on the regions of the target objects instead of being distracted by the background or other irrelevant objects.

In the work of integrating the GAM and DLKA mechanisms into YOLOv8s, respectively, for the surface defect detection of carbon fiber unidirectional tape prepregs, each demonstrates unique innovation:

##### Innovation of the GAM

Differentiation between Background Texture and Defects: The surface of carbon fiber prepregs has a vertical periodic texture background, and the characteristics of various defects are significantly different. The GAM is precisely integrated between the backbone network and the detection head, which can sort out and integrate the global texture information. In this way, when dealing with fiber tangles defects, the GAM can grasp the overall image and use its global information capture ability to quickly locate fiber tangles in the complex periodic texture background. Since the fiber tangles differ from the background texture in terms of morphology and structure, the GAM can highlight their unique agglomerated form, avoid background interference, and improve the detection accuracy.

Optimization of Edge Defect Detection: Considering that the resin-rich defects in the dataset often appear in the edge areas of the samples, the GAM can focus on the global feature changes in the edge areas when processing images. Even if the difference between the resin-rich area and the background is small, the GAM can effectively detect resin-rich defects by integrating the global edge information.

Complementary Collaboration with the Original Model: The addition of the GAM forms a complementary relationship with the original feature extraction and detection mechanism of YOLOv8s. The original model focuses on local feature extraction and conventional target detection, while the GAM focuses on global information integration. When detecting surface defects of carbon fiber prepregs, the two work together. For example, when detecting wrinkle defects, the original model extracts local texture change features, and the GAM judges the overall structural changes of the fabric from a global perspective, jointly improving the detection performance of wrinkle defects.

##### Innovation of the DLKA Mechanism

Breakthrough in Concealed Defect Detection: Fiber splits defects appear as thin slits, and their directions are consistent with the background texture. The DLKA is good at capturing subtle local features and directional information. The deformable large kernel of the DLKA can adaptively adjust the size and shape of the receptive field according to the characteristics of fiber splits defects, flexibly covering the fiber splits area. It can accurately extract the subtle features of fiber splits, solving the problem of easy missed detection.

Capture of Variable Defect Features: Given that wrinkle defects vary in size and shape, the deformable large kernel of the DLKA can dynamically adjust the receptive field according to the actual shape and size of the wrinkles, comprehensively extracting the features of the wrinkles. Whether it is a small wrinkle or a complex large wrinkle, the DLKA can accurately capture its local features, such as the texture change at the fold and the undulating edge, improving the detection ability of wrinkle defects.

Enhanced Detection of Local Details: The addition of the DLKA introduces a powerful local detail detection ability to the model. In the detection process, the DLKA focuses on the local area of the image and conducts in—depth analysis of the subtle features of the defects. For example, when detecting fiber tangles, it can accurately extract the details of fiber entanglement, which helps to more accurately judge the severity of fiber tangles and enriches the detection dimensions of the model for defects.

Handling of Complex Defect Scenarios: For various complex defect scenarios that may occur on the surface of carbon fiber prepregs, such as the co-existence of multiple defects or the interweaving of defects and background interference, the DLKA can accurately identify defect features in a complex local environment with its flexible receptive field adjustment ability. For example, in the complex area where fiber splits may exist at the edge of a resin-rich defect, DLKA can adjust the receptive field according to different defect features respectively, achieving effective detection of multiple defects in complex scenarios and expanding the application scope of the model.

### 2.3. Experimental Design

#### 2.3.1. Neural Network Architecture Setup

The acquired prepreg images, with a size of 4096 × 4000, were too large. Thus, the input image size was compressed to 1280 × 1280. The input had 3 channels (RGB). The batch size for each training batch was set to 8.

**Figure 8:**
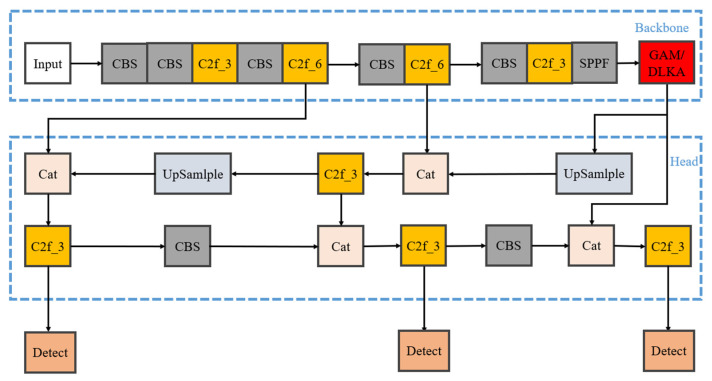
Improved YOLOv8s network architecture with attention mechanisms.

Model training was conducted for the original YOLOv8s, the YOLOv8s added with the GAM, and the YOLOv8s added with DLKA, individually. The architectures of the models with attention mechanisms are shown in Figure 8. The training was performed for 400 epochs.

The main reasons for inserting the attention mechanism module between the backbone network and the detection head are as follows: Both the backbone network and the detection head have a high level of encapsulation. The backbone network is responsible for feature extraction, while the detection head is tasked with outputting the detection results based on the extracted features. The structures of these two components have been verified to be highly effective over a long period. Arbitrary modifications are highly likely to reduce the performance of the model. Inserting a module between them will not affect the well-encapsulated original structure. Moreover, it can enable the model to better focus on the features that need to be extracted after the feature extraction process and before the output of the detection results, thereby improving the performance of the model.

#### 2.3.2. Evaluation Metrics

This study employs precision (*P*), recall (*R*), mean average precision (mAP), required floating-point operations per second (GFLOPS), model size, and single-frame inference time to evaluate model performance.

mAP refers to the calculation of the average precision for each defect category first, and then the comprehensive average precision of all categories. Generally, it refers to mAP@0.5, which is the mean average precision with an IoU threshold of 0.5. The specific calculation formulas for *P*, *R*, and mAP are as follows:

(1) $$P=TP/\left(TP+FP\right)$$

(2) $$R=TP/\left(TP+FN\right)$$

(3) $$AP=\int_{0}^{1}P(R)dR$$

(4) $$\mathrm{mAP}=\frac{\sum_{i=1}^{N}{AP}_{i}}{N}$$

In the formulae, *N* represents the number of detection categories, which is 5. *TP* stands for true positive samples, referring to the number of correctly identified defects, specifically determined when the predicted box and the labeled box have the same category and the IOU is greater than 0.5. *FP* indicates false positive samples, which is the number of defects wrongly identified. *FN* represents false negative samples, denoting the number of defects that were not identified.

## 3. Results and Analysis

**Figure 9:**
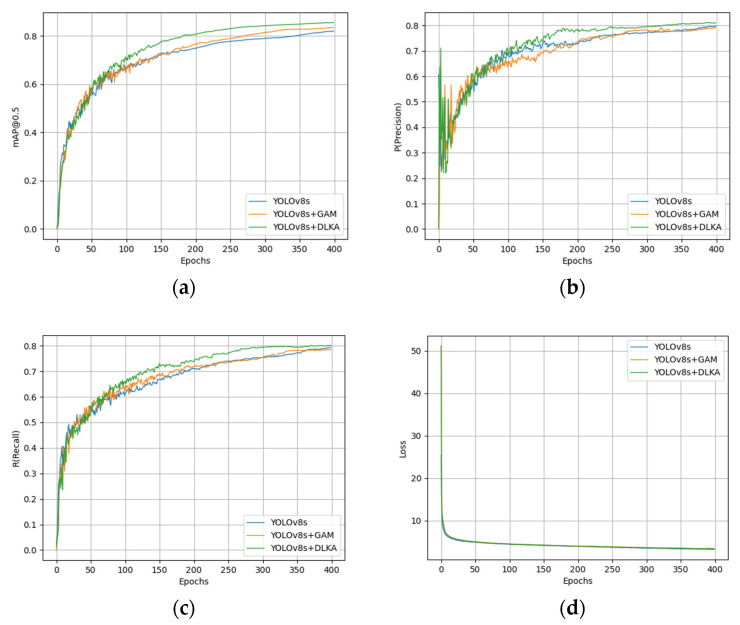
Evolution of metrics during training for YOLOv8s, YOLOv8s-GAM, and YOLOv8s-DLKA: (**a**) mAP@0.5; (**b**) precision; (**c**) recall; (**d**) loss.

To validate the effectiveness of incorporating attention mechanisms, this study conducted comparative analysis on the original YOLOv8s model, the YOLOv8s model with the GAM, and the YOLOv8s model with DLKA. As shown in Figure 9, the curves of mAP@0.5, precision (*P*), recall (*R*), and total loss during the training process are presented for all models.

During the first 50 epochs, both the baseline model (YOLOv8s) and the variants with added attention mechanisms (GAM/DLKA) exhibited nearly identical growth rates in mAP@0.5, their performance trajectories having been heavily influenced by pre-trained weights during the initial stages. Beyond the 50th epoch, models incorporating attention mechanisms demonstrated faster convergence speeds and a more focused optimization on critical features.

Throughout the initial phase, precision metrics remained comparable between the original and attention-enhanced models. Post-50 epochs, the YOLOv8s-DLKA variant achieved a sharper rise in precision—significantly reducing false positives and improving detection specificity. Additionally, this recall of the model improved more rapidly, highlighting role of attention mechanisms in amplifying detection coverage and minimizing missed detections (false negatives). These observations collectively validate efficacy of attention modules in enhancing both localization accuracy and class discrimination in target detection tasks.

**Figure 10:**
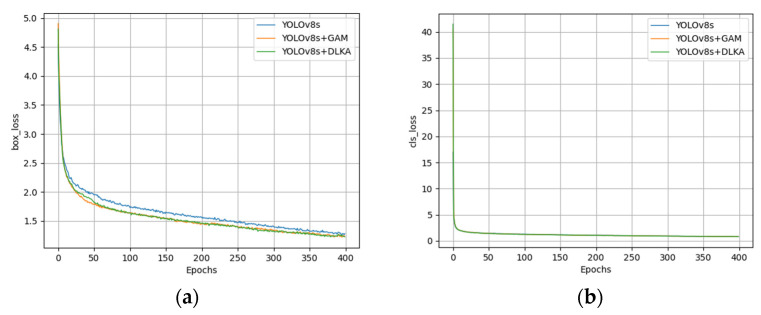
Curves of bounding box loss function and classification loss function during training of YOLOv8s, YOLOv8s-GAM, and YOLOv8s-DLKA models: (**a**) Bounding Box Loss; (**b**) Classification Loss.

Figure 10 presents the training curves of bounding box loss and classification loss functions for YOLOv8s, YOLOv8s-GAM, and YOLOv8s-DLKA models. The results demonstrate that attention-enhanced models (GAM/DLKA) achieved a significantly faster decline in bounding box loss compared to the baseline YOLOv8s, indicating enhanced spatial localization capability. However, classification losses showed no marked distinction across all variants, suggesting that attention mechanisms primarily optimize object position refinement rather than category discrimination. This spatial precision improvement directly correlates with the ability of attention modules to better localize targets, thereby notably boosting accuracy in pinpointing defect locations.

Table 1 summarizes the precision and recall values of YOLOv8s, YOLOv8s-GAM, and YOLOv8s-DLKA models. The improved models demonstrate enhanced both precision (reducing false positives) and recall (reducing false negatives), thereby decreasing the likelihood of model errors such as incorrect detections and missed targets.

Table 2 presents the mAP@0.5 values of various defect types evaluated across different models. For the fiber split defect, the model incorporating the DLKA module achieves a 19.7% increase in mAP@0.5 (compared to the baseline YOLOv8s). The overall mAP@0.5 averaged across all defect categories improves by 3.6%, significantly boosting detection accuracy and robustness.

Table 3 lists the performance metrics of various models. The models equipped with an attention mechanism require greater computational resources and more parameters, and they increase inference time. However, the maximum single-frame inference time remains at 58 ms, retaining a high inference speed, while simultaneously achieving significant improvements in computational precision.

**Figure 11:**
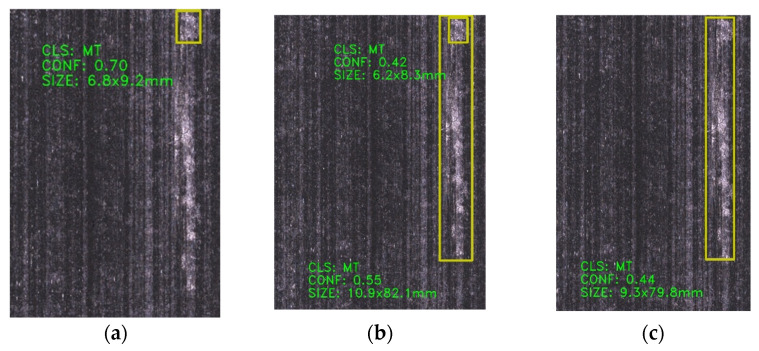
Comparison of detection results for the same fiber tangles defect image across different models: (**a**) YOLOv8s, (**b**) YOLOv8s-GAM, (**c**) YOLOv8s-DLKA.

Figure 11a–c compares detection results of a fiber tangles defect image using various YOLO models. Whereas Figure 11a, derived from the original YOLOv8s model, successfully identifies defect locations but fails to fully enclose the defect with bounding boxes, the improved YOLOv8s models (Figure 11b,c) demonstrate enhanced target localization—this aligns with the accelerated convergence of the bounding box loss function observed in prior experiments.

**Figure 12:**
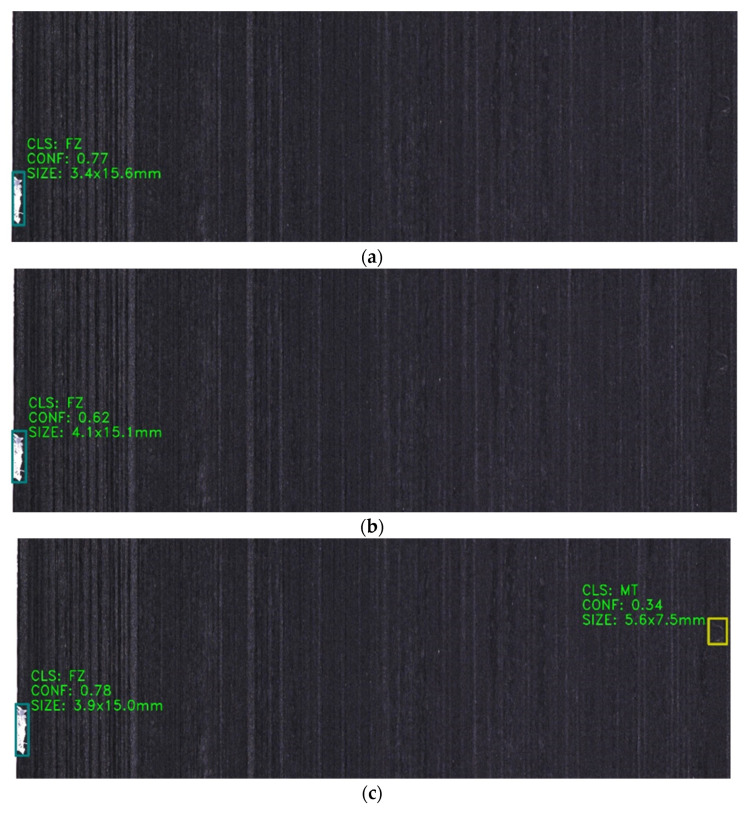
Comparison of detection results for the same resin-rich defect image across different models: (**a**) YOLOv8s, (**b**) YOLOv8s-GAM, (**c**) YOLOv8s-DLKA.

**Figure 13:**
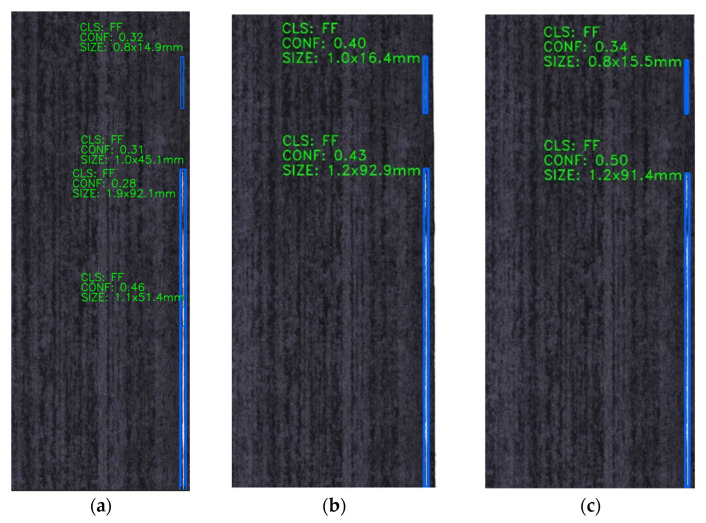
Comparison of detection results for the same fiber splits defect image across different models: (**a**) YOLOv8s, (**b**) YOLOv8s-GAM, (**c**) YOLOv8s-DLKA.

For a resin-rich defect image (Figure 12), the enhanced model (Figure 12c) elevates detection confidence scores while identifying targets missed by the baseline YOLOv8s. Figure 13 evaluates fiber splits defects, revealing that the original model (Figure 13a) exhibits repetitive detections, whereas the improved variants (Figure 13b,c) significantly reduce both false negatives and repetitive false positives, addressing the key of original model limitations.

**Figure 14:**
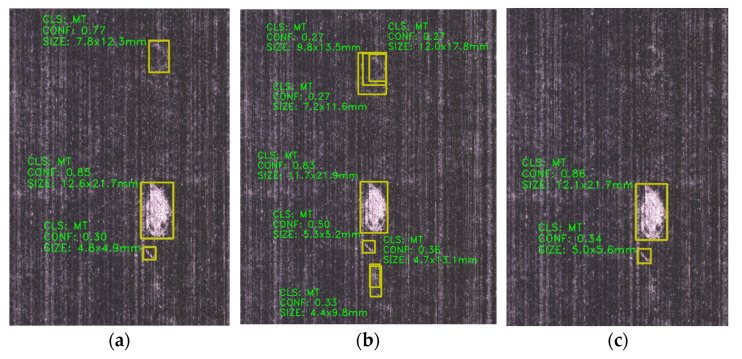
Comparison of detection results for the same fiber tangles defect image across different models: (**a**) YOLOv8s, (**b**) YOLOv8s-GAM, (**c**) YOLOv8s-DLKA.

Lastly, Figure 14 compares detection results of the same fiber tangles defect: Figure 14a shows false positives in the original model, while Figure 14b, using the YOLOv8s-GAM variant, still produces misclassifications and redundant bounding boxes. In contrast, the YOLOv8s-DLKA model (Figure 14c) achieves precise defect localization without false positives/negatives, empirically validating the effectiveness of the DLKA mechanism.

Prepreg material products have a width of 1000 mm. Considering the overlapping areas between cameras, each camera used for imaging has a field of view (FOV) width of 280 mm. The required horizontal camera resolution (in pixels) is calculated as

Resolution (pixels) = FOV (280 mm)/Detection Precision. To meet the minimum resolution requirement of 2800 pixels, the system adopts 4K linear array cameras. The 4K linear array camera features a 4096-pixel resolution, 7 μm pixel pitch, and a 28.672 mm sensor target surface.

Calculation of pixel precision:

Pixel Resolution = FOV (280 mm)/Effective Pixels (4090 pixels) = 0.06835 mm/pixel. This means each pixel corresponds to 0.06835 mm in actual dimensions. The detection results demonstrate that the system can identify defects as small as 0.8 mm in size.

**Figure 15:**
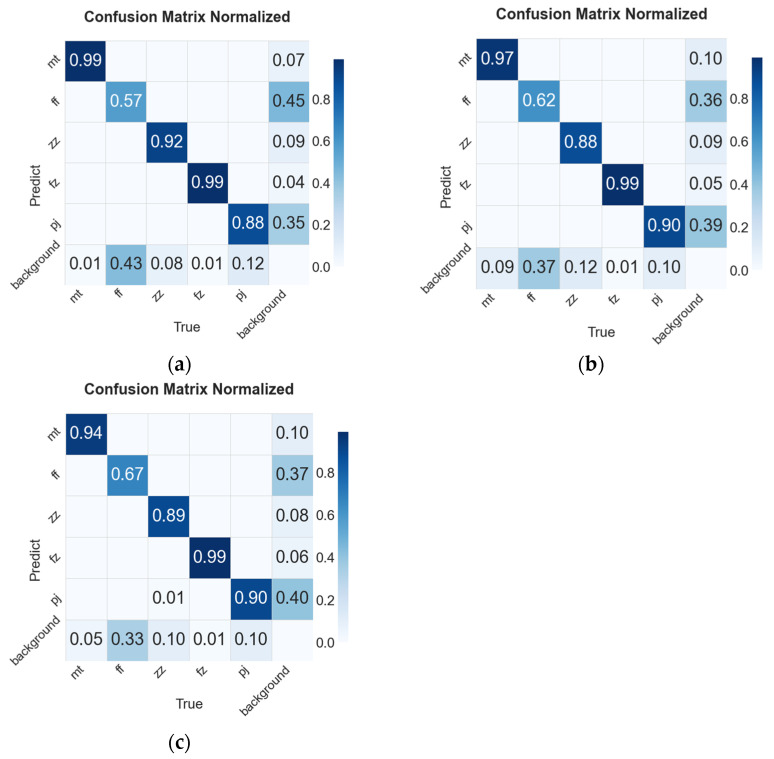
The normalized confusion matrices of different models: (**a**) YOLOv8s, (**b**) YOLOv8s-GAM, (**c**) YOLOv8s-DLKA.

Figure 15 presents the normalized confusion matrices of different models. In particular, concerning the fiber splits defects, it can be observed that for the original YOLOv8s model, the probability of classifying an actual fiber splits defect as the background is 0.43. For the YOLOv8-GAM model, this probability of misclassifying an actual fiber splits defect as the background is 0.37. As for the YOLOv8-DLKA model, the probability of misidentifying an actual fiber splits defect as the background is 0.33. The results suggest that the improved models have effectively reduced the probability of missed detections.

**Figure 16:**
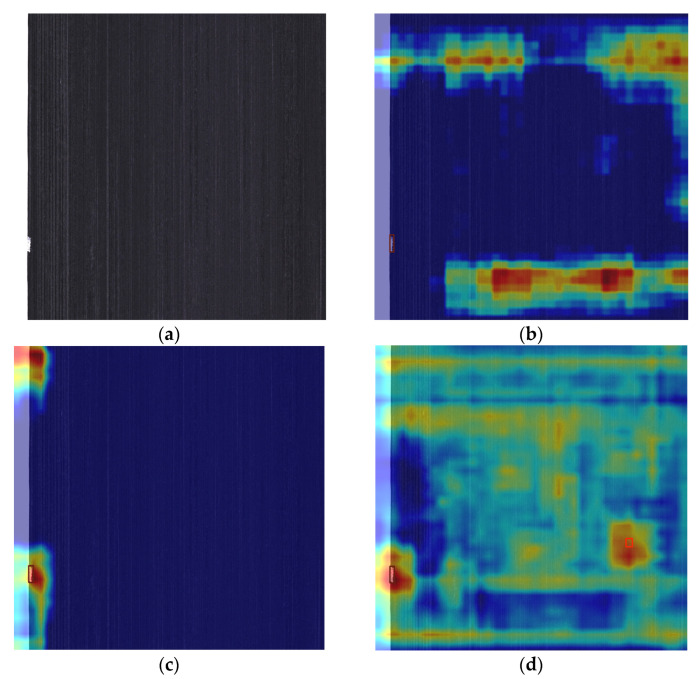
Original images and attention heatmaps under different models: (**a**) Original images (**b**) YOLOv8s, (**c**) YOLOv8s-GAM, (**d**) YOLOv8s-DLKA.

To more intuitively observe the improvement in the defect recognition ability of the improved model, this study employs the Grad-CAM (gradient weighted class activation mapping) technique to draw heatmaps, which allows for a relatively intuitive visualization of how the model learns defect features. Grad-CAM performs backpropagation using the training weights, executes global average pooling on the gradient matrix in the spatial dimension, and conducts weighted activation processing on each channel of the feature layer, thereby generating a visualized heatmap. In the heatmap, the brightness of each region can reveal the feature regions in the image that have a significant impact on the prediction results of the models. The comparison of heatmap visualizations is shown in Figure 16.

Compared with the YOLOv8s model, the defect targets in the YOLOv8s-GAM and YOLOv8s-DLKA models exhibit brighter colors and higher response levels. The enhanced perception of the correct targets enables the models to focus more accurately on the features of the defect regions.

## 4. Conclusions

This study proposed an improved version of the YOLOv8s model with attention mechanisms. Through comparative analysis of results, the following conclusions were drawn:

Under identical experimental conditions, the YOLOv8s-GAM model achieved a mAP@0.5 of 84.4%, with a precision of 79.3% and recall of 78.3%. The YOLOv8s-DLKA model demonstrated a mAP@0.5 of 86.4%, precision of 82.4%, and recall of 80.2%. Compared to the original YOLOv8s model, the improved models achieved 1.6% and 3.6% improvements in mAP@0.5, 0.9% and 4.1% gains in precision, and 1.4% and 3.6% enhancements in recall, respectively.

To validate the detection effectiveness, this study conducted visual comparisons of defect detection on the same defect image using different models. The improved models enhanced target localization accuracy, reducing false negatives and false positives. In industrial inspection, false negatives are far more critical and intolerable than false positives. The proposed models significantly reduced the likelihood of missed detections, which is of critical significance for practical industrial quality control.

Nonetheless, the proposed models still face challenges in detecting certain defect types, leaving room for further refinement. Future work will explore novel modules to optimize detection performance and enhance the practical applicability of this method. In addition, we will consider making lightweight improvements to the network structure to enhance the inference speed, modify the loss function of the network to achieve higher boundary localization performance, and use two-stage object detection algorithms such as Faster R-CNN for model training. This research contributes to advancing industrial anomaly detection and improving quality assurance for manufacturing enterprises.

## Figures and Tables

**Table 1:** Precision and recall of YOLOv8s, YOLOv8s-GAM, and YOLOv8s-DLKA models.

Model	Precision	Recall
YOLOv8s	78.3%	76.9%
YOLOv8s-GAM	79.2%	78.3%
YOLOv8s-DLKA	82.4%	80.5%

**Table 2:** mAP@0.5 values for various defect types across YOLOv8s, YOLOv8s-GAM, and YOLOv8s-DLKA models.

Model	Fiber Tangles	Fiber Splits	Wrinkles	Resin-Rich	Resin-Poor
YOLOv8s	97.4%	48.2%	88.5%	98.9%	81.2%
YOLOv8s-GAM	95.0%	57.8%	85.4%	98.8%	85.2%
YOLOv8s-DLKA	91.9%	67.9%	86.7%	98.6%	87.0%

**Table 3:** Performance metrics of YOLOv8s, YOLOv8s-GAM, and YOLOv8s-DLKA models.

Model	Parameters/10^6^	FLOPs/G	Single-Frame Inference/ms	mAP@0.5	mAP@[0.5:0.95]
YOLOv8s	9.84	23.6	14.4	82.8%	54.8%
YOLOv8s-GAM	17.28	32.6	23.4	84.4%	52.9%
YOLOv8s-DLKA	13.22	29.3	58.0	86.4%	55.4%

## Data Availability

Data are contained within the article.

## Abbreviations

The following abbreviations are used in this manuscript:

AVIC	Aviation Industry Corporation of China
YOLO	You only look once
GAM	Global Attention Mechanism
DLKA	Deformable Large Kernel Attention
